# SEOM Clinical Guideline for the treatment of pancreatic cancer (2016)

**DOI:** 10.1007/s12094-016-1586-x

**Published:** 2016-11-28

**Authors:** R. Vera, E. Dotor, J. Feliu, E. González, B. Laquente, T. Macarulla, E. Martínez, J. Maurel, M. Salgado, J. L. Manzano

**Affiliations:** 1 Department of Medical Oncology, Complejo Hospitalario de Navarra, c/Irunlarrea-3, 31008 Pamplona, Spain; 2 Consorcio Sanitario de Terrassa, Barcelona, Spain; 3 Hospital Universitario la Paz, Madrid, Spain; 4 Complejo Hospitalario Universitario de Granada Virgen de las Nieves, Granada, Spain; 5 ICO-Hospitalet de LLobregat, Hospital Duran i Reynals, Hospitalet de Llobregat, Spain; 6 Hospital Vall`Hebron, Barcelona, Spain; 7 Hospital Universitario Marqués de Valdecilla, Santander, Spain; 8 Hospital Clínic i Provincial de Barcelona, Barcelona, Spain; 9 Complexo Hospitalario de Orense (CHUO), Ourense, Spain; 10 ICO-Badalona, Hospital Germans Trias i Pujol, Barcelona, Spain

**Keywords:** Pancreatic cancer, Treatment, Diagnostic, Guidelines

## Abstract

Pancreatic cancer remains an aggressive disease with a 5 year survival rate of 5%. Only 15% of patients with pancreatic cancer are eligible for radical surgery. Evidence suggests a benefit on survival with adjuvant chemotherapy (gemcitabine o fluourouracil) after R1/R0 resection. Adjuvant chemoradiotherapy is also a valid option in patients with positive margins. Borderline resectable pancreatic cancer is defined as the involvement of the mesenteric vasculature with a limited extension. These tumors are technically resectable, but with a high risk of positive margins. Neoadjuvant treatment represents the best option for achieving an R0 resection. In advanced disease, two new chemotherapy treatment schemes (Folfirinox or Gemcitabine plus nab-paclitaxel) have showed improvements in overall survival compared with gemcitabine alone. Progress in pancreatic cancer treatment will require a better knowledge of the molecular biology of this disease, focusing on personalized cancer therapies in the near future.

## Introduction

Pancreatic cancer is a major health problem. Despite not having a high incidence in the population, its aggressive behavior leads to a high mortality [1]. In Spain, 6367 new cases were diagnosed in 2012 (3% of global cancer incidence), 3335 in men and 3032 in women. Pancreatic cancer was the fourth most fatal cancer (5720 cases) in Spain, after lung, colorectal, and breast cancer. In 2012, approximately 3000 men and 2700 women died from this disease [2]. Most patients who are diagnosed with pancreatic cancer are aged between 65 and 70 years old. It is rare that this tumor appears below 60 years of age; in this case, we should rule out the association with a genetic alteration. Only a small proportion of pancreatic cancers are related to a genetic alteration (5–10%). Germline mutations in BRCA2, p16, ATM, STK11, PRSS1/PRSS2, SPINK1, PALB2, and DNA mismatch repair genes are associated with varying degrees of increased risk for pancreatic carcinoma. Mutation in BRCA2 is probably the most common inherited disorder in familial pancreatic cancer [3]. Among the remaining 90%, the major risk factors are tobacco, *H. pylori* infection, and factors related to dietary habits [4].

At the time of diagnosis, less than 20% of patients are suitable for resection, given the advanced stage of the disease. After surgical resection, survival rates usually are between 10 and 20 months. Defining the treatment strategy for patients suffering from pancreatic carcinoma requires a specialized multidisciplinary team.

## Diagnosis and staging

### Diagnosis

After a suspicion of pancreatic cancer based on signs and symptoms (weight loss, jaundice, pain or depression, among others), pathologic diagnosis is mandatory in unresectable and borderline resectable cases in which a preoperative treatment is planned.

A pathological diagnosis of PC is usually made with fine-needle aspiration (FNA) by endoscopic ultrasound (EUS) guidance or computed tomography (CT). EUS-FNA is preferred in cases of resectable and borderline resectable disease. Cytologic specimens have limitation for biomarkers studies and do not include stroma. Core needle biopsies (CNB), that use a slightly larger and hollow needle to withdraw small cylinders of tissue, could be more useful in the near future.

Diagnosis of pancreatic cancer should include cytologic or pathologic diagnosis [5], staging (Table 1), and evaluation of the patient basal situation (PS, comorbidities…) and his preferences. Unnecessary delays should be avoided to treat the patient as soon as possible.

**Table 1:** Evaluations recommended for the proper staging of pancreatic cancer

Complete history and physical examination
Laboratory test: blood count and serum chemistry including PCR, albumin and levels of the carbohydrate antigen CA19-9
CT of the chest and abdomen
Histologic or cytologic diagnosis
Bone scan in presence of bone pain, elevated serum calcium or elevated alkaline phosphatase levels
In patients with resectable tumors (optional), border-line or locally advanced pancreatic cancer (mandatory)
EUS+FNA
In patients with borderline resectable tumors
Diagnostic laparoscopy will be assessed in cases of suspicion of peritoneal involvement (no consensus)

### Staging system

The classification system most frequently used in pancreatic cancer is the tumor-node-metastasis (TNM) system of the combined American Joint Committee on Cancer (AJCC)/International Union Against Cancer (UICC). This staging system classifies tumors depending on the size and extent of the primary tumor (T), the presence or absence of regional lymph node metastasis (N) and the presence or absence of distant metastasis (M). The latest update is the 8th edition of this classification system published in 2016 and recently validated (Table 2) [6].

**Table 2:** Staging group

Primary tumor (T)
T1: Maximum tumor diameter ≤2 cm
T2: Maximum tumor diameter >2 ≤ 4 cm
T3: Maximum tumor diameter >4 cm
T4: Tumor involves the celiac axis or the superior mesenteric artery (unresectable primary tumor)
Regional lymph nodes (N)
N0: No regional lymph node metastasis
N1: Metastasis in 1–3 regional lymph nodes
N2: Metastasis in 4 regional lymph nodes
Distant metastases (M)
M0: No distant metastasis
M1: Distant metastasis

Stage	T	N	M
0	Tis	N0	M0
IA	T1	N0	M0
IB	T2	N0	M0
IIA	T3	N0	M0
IIB	T1–3	N1	M0
III	Any T T4	Any N	M0
IV	Any T	Any N	M1

All patients with PC should be valued from the beginning at a multidisciplinary committee in a reference center with an adequate volume of patients, for decision-making regarding treatment, especially those with potential surgical indication. It is available a classification that allows to evaluate the potential resectability based on radiological findings (Table 3) [7, 8].

**Table 3:** Criteria defining resectability status according to NCCN Guidelines version 1.2016 (Pancreatic adenocarcinoma)

Resectability status	Distant metastases	Arterial	Venous
Resectable	No	No arterial tumor contact [celiac axis (CA), superior mesenteric artery (SMA) or common hepatic artery (CHA)]	No tumor contact with the superior mesenteric vein (SMV) or portal vein (PV) or ≤180° contact without vein contour irregularity
Boderline resectable	No	Head/uncinated process: Solid tumor contact with CHA without extension to CA or hepatic artery bifurcation allowing for safe and complete resection and reconstruction Solid tumor contact with the SMA of ≤180° Body and tail: Solid tumor contact with the CA of ≤180° Solid tumor contact with the CA of >180° without involvement of the aorta and with intact and uninvolved gastroduodenal artery	Solid tumor contact with the SMV or PV of >180°, contact of ≤180° with contour irregularity of the vein or thrombosis of the vein but with suitable vessel proximal and distal to the site of involvement allowing for safe and complete resection and vein reconstruction Solid tumor contact with the inferior vena cava (IVC)
Unresectable	Yes (including non-regional lymph node metastasis)	Head/uncinated process: Solid tumor contact with SMA >180° Solid tumor contact with CA >180° Solid tumor contact with the first jejunal SMA branch Body and tail: Solid tumor contact of >180° with de SMA or CA Solid tumor contact with the CA and aortic involvement	Head/uncinated process: Unreconstructible SMV/PV due to tumor involvement or occlusion (can be due tumor or bland thrombus) Contact with most proximal draining jejunal branch into SMV Body and tail: Unreconstructible SMV/PV due to tumor involvement or occlusion (can be due tumor or bland thrombus)

### Recommendations

- Laboratory test with CA19-9, CT chest and abdomen, histologic or cytologic diagnostic, EUS in resectable tumors (IV, C).

## Treatment

### Resectable disease

Surgery is the standard treatment for resectable disease (70% of patients have positive margins independently of the quality of the surgical resection).

Patients with tumors located in the pancreatic head are treated with pancreatoduodenectomy (Whipple procedure). When the tumor is located in the body or tail of the gland, the surgical procedure is a distal pancreatectomy. In some cases, a total pancreatectomy may be required.

Even with a R0 resection, the recurrence rate is very high. Therefore, adjuvant treatment is required in almost all the patients with resected adenocarcinoma of the pancreas. It is advisable to start adjuvant therapy between 6 and 8 weeks after surgery.

Post-operative treatment in pancreatic cancer has been evaluated in several clinical trials.

CONKO-1 trial demonstrated that patients treated with adjuvant gemcitabine (1000 mg/m^2^ day 1, 8, 15/28 days) for 6 months after surgery presented longer disease-free survival than those patients treated with surgery alone (13.4 vs. 6.9 months, *p* < 0.001) [9]. The ESPAC-3 trial compared the administration of adjuvant chemotherapy with 6 months of either bolus fluorouracil (425 mg/m^2^ and folinic acid 20 mg/m^2^ day 1–5 every 28 days) or gemcitabine. Median survival was equivalent in both arms (23 months, *p* 0.39), but gemcitabine was better tolerated [10].

As a result of these studies, gemcitabine and 5-fluorouracil can both be considered as the standard of adjuvant treatment in pancreatic cancer.

This year in the last ASCO meeting, preliminary results of ESPAC-4 trial were presented. Adjuvant gemcitabine at a standard dose was compared with the combination of gemcitabine (1000 mg/m^2^ day 1, 8, 15/28 days) and capecitabine (1660 mg/m^2^/day d1–21/28 days) for 6 months [11]. Median survival for patients treated with gemcitabine and capecitabine was significantly better than gemcitabine monotherapy (28 vs. 25.5 months, HR 0.82, *p* 0.032). The role of chemoradiotherapy has not been clearly established as beneficial because of the controversial results in previous studies, although it is an option in some individual cases with R1 resection [12].

### Recommendations

1. Adjuvant treatment is required in all resected patients if the patient is recovered from the surgery (I, A).
2. Six cycles of gemcitabine or 5-fluorouracil is considered a standard treatment (I, A).

## Borderline resectable disease

Numerous non-randomized studies in patients with marginally resectable disease that have used different treatment regimens with neoadjuvant chemotherapy or chemoradiation have managed to increase the resectability rate from 33 to 64% in borderline tumors. There is limited evidence to recommend specific neoadjuvant regimens off-study, and practices vary with regard to the use of chemotherapy and chemoradiation [13, 14].

The benefit of neoadjuvant therapies in borderline resectable pancreatic cancer has been retrospectively reviewed by different authors, with treatment regimens including 2–4 months of neoadjuvant chemotherapy followed by radiation in combination with 5-fluorouracil (5-FU), gemcitabine, capecitabine, or paclitaxel.

Some studies have evaluated the feasibility and efficacy of FOLFIRINOX or gemcitabine + albumin-bound paclitaxel for neoadjuvant treatment with a small number of patients [15].

### Recommendations

- Patients with borderline resectable lesions should be included in clinical trials wherever possible. Acceptable regimens include FOLFIRINOX or gemcitabine + albumin-bound paclitaxel (III, B).
- Chemoradiation with gemcitabine or capecitabine-based regimens are another option (III, C).

## Locally advanced unresectable disease

The objective in unresectable locally advanced pancreatic cancer (LAPC) is to increase the survival and quality of life, maintaining local control of the disease. Complications of tumor growth may require decompression maneuvers of the digestive tract or bile duct prior to an active cancer treatment. This must always be accompanied by an appropriate treatment of symptomatic support, initiated at the time of diagnosis [16]. The initial approach is controversial. Radiochemotherapy (RT-QT) improves survival compared to best supportive care or radiotherapy (RT) alone, but with increased toxicity [17]. The LAP07 study showed no benefit in survival when comparing RT-QT vs. chemotherapy (CT) in patients with LAPC and controlled after 4 months of induction QT treatment disease, but showed better local control and increased free survival progression [18]. The standard QT treatment in LAPC is gemcitabine, but the significant increase in efficacy with new schedules in metastatic disease (FOLFIRINOX [19] and gemcitabine/nab-Paclitaxel [20]) has lead to their use, also in LAPC, as a reasonable alternative in patients with good performance status (ECOG PS 0–1).

The duration of the initial treatment is not established and depends on tolerability and tumor response.

Using chemoradiotherapy consolidation in patients with response or stabilization after 4–6 months of chemotherapy is an option to consider, vs. maintenance treatment with chemotherapy in patients with good PS. In this context, capecitabine showed a better toxicity profile and efficacy than gemcitabine [21].

### Recommendations

1. The standard treatment for LAPC is chemotherapy with gemcitabine (I, A).
2. By extrapolation of phase III studies in metastatic disease, FOLFIRINOX or gemcitabine/nab-Paclitaxel represents a valid alternative in patients with PS 0–1 and suitable profile of comorbidity (V, B).
3. After stabilization QT response or induction (4–6 months) consolidation with RT-QT is an alternative to maintaining QT in selected patients (II, B).

## Metastatic disease

In patients with pancreatic carcinoma (PC), it is crucial to evaluate patient-related and disease-related characteristics, such as the intensity of pain (with analogic scales), the analgesic requirements, loss of weight (in percentage), the ECOG scale of performance status, the Glasgow Modified Score (that include albumin and protein reactive C), and a geriatric assessment in patients >70 years. The geriatric assessment should include comorbidities, cognition, mental health status and support, fatigue, the assessment of polypharmacy, and the presence of geriatric syndromes. Since elderly patients represent a very heterogeneous group, this complex assessment will help to define the frailty or fitness and to guide treatment decisions.

Until 2011, gemcitabine has been considered as the standard of care for patients with metastatic pancreatic cancer [22].

**Fig. 1:**
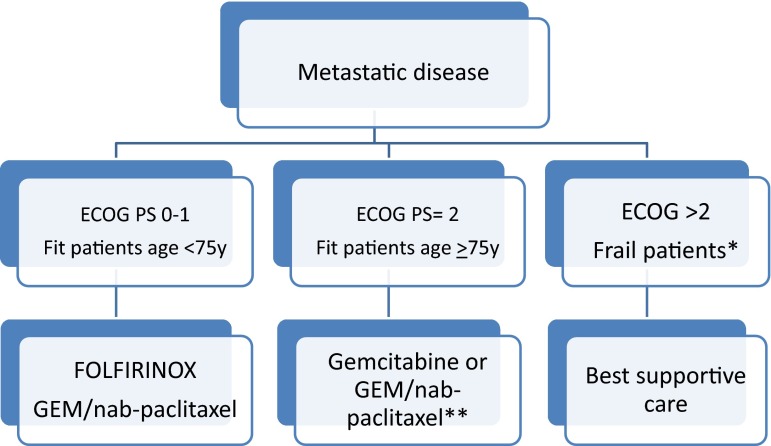
*Comorbidities, cognition, mental health status and support, fatigue, assessment of polypharmacy, and the presence of geriatric syndromes. **The efficacy of GEM/nab-paclitaxel over gemcitabine in this specific subgroup of patients is currently insufficient

The intense chemotherapeutic regimen of 5-FU, oxaliplatin, and irinotecan (FOLFIRINOX) has been shown superior to GEM monotherapy in PFS and OS [19]. PC patients with mutation in BRAC1, BRAC2 or PALB2 with a prevalence of 10% would be especially sensitive to this combination, an issue that should be tested prospectively [23]. In the MPACT phase III clinical trial, the combination of gemcitabine and nab-paclitaxel also demonstrated superiority in terms of efficacy for metastatic pancreatic cancer compared with gemcitabine monotherapy [20]. FOLFIRINOX and GEM/nab-paclitaxel are both indicated in fit PC patients with ECOG PS 0, 1 and <75 years old. In fit elderly patients (>75 years) or patients with performance status 2, single-agent GEM can be recommended, though in selected patients, gemcitabine and nab-paclitaxel can be considered (Fig. 1). There is no standard chemotherapy for second-line treatment. In patients progressing to gemcitabine or gemcitabine-based combinations, 5-FU/oxaliplatin or 5FU/liposomal irinotecan (Nal-Iri) combination would be considered in selected patients with good PS [24, 25]. It should be noted that none of these schedules has proven efficacy, in patients progressing to FOLFIRINOX or GEM/nab-paclitaxel. PD1 and PD-L1 inhibitors have not shown activity in PC. PC is a paradigmatic example of a weak immunogenic tumor due to the imbalance between a poor lymphocyte infiltrate (especially CD8) and a high stromal presence that induce a TH2 polarization [26, 27]. Combination of PD-1 or PD-L1 with vaccines or drugs targeting the stroma (either macrophages or fibroblast) deserves to be tested in selected PC patients.

### Recommendations

1. FOLFIRINOX and/or Gem-Abraxane are standard first-line schedules in metastatic disease in patients with ECOG/PS 0–1 (I, A).
2. In selected patients with ECOG 2, gemcitabine and nab-paclitaxel can be considered (II, B).
3. Gemcitabine is an option in elderly patients or patient with PS2 (I, B).

## Supportive care

Patients with pancreatic cancer may experience various distressing symptoms that require ongoing supportive care from diagnosis through treatment (either curative or noncurative). Initial pain management may involve non-opioid drugs, but the mainstay of pain management typically is opiate medication. Celiac plexus block may be used to improve pain relief. It can be performed via percutaneous under ultrasound control, surgical or endoscopic [28]. Patients merit a consultation with an endocrinology or nutritionist because of anorexia and weight loss. Some patients experience exocrine or endocrine pancreatic insufficiency and require pancreatic enzyme replacement and/or insulin therapy [28]. In case of biliary obstruction, the preferred treatment is endoscopic placement of a metal permanent self-expanding stent in the bile duct. Plastic stents can be considered for patients expected to survive <4 months [29]. Gastric outlet/duodenal obstruction occur in up to 10% of patients with pancreatic cancer. Endoscopic duodenal stenting can be successful in the majority of these patients, and median duration of stent patency is 6 months [29]. If venous thromboembolism is presented, in selected high-risk patients (p.e Khorana Index ≥3) and no risk of bleeding, prophylaxis with low molecular weight heparin could be considered on a case-by-case basis [30].

### Recommendations

1. Pain management typically is opiate medication (I, A).
2. Biliary and duodenal obstruction can be managed successful with endoscopic stent placement (I, A).

## Follow-up

Five-year survival rate after surgical resection of PC is 15–25% and correlates with resection margin status (R0 vs. R1) and lymph node metastases. The majority of recurrences occur within 2 years after resection and can be locoregional and/or to distant sites, including the liver, lung, or peritoneum [31]. Although computed tomography, positron emission tomography and the serum CA19-9 can detect preclinical recurrences, limited available information does not support the idea that early treatment of relapse improves survival [32]. Therefore, considering the poor prognosis of the disease upon diagnosis of a recurrence, there is no evidence that regular follow-up has any impact on the outcome. Follow-up visits should concentrate on symptoms, nutrition, and psycho-social support, resolution of treatment-related toxicity, addressing survivorship issues and monitoring for recurrence [33].

### Recommendations

- There is no evidence that regular follow-up after initial therapy with curative intent is useful (IV, D).
